# Four-domain gut metagenomics reveals archaeal-centered cross-kingdom remodeling across coronary artery disease and acute myocardial infarction

**DOI:** 10.1016/j.isci.2026.117227

**Published:** 2026-08-25

**Authors:** Yin Yue, Wanyu Wei, Chenpeng Wu, Ni Suo, Zhengwei Zhang, Wenting Liu, Qing Su, Meixin Wang, Yongqing Zhang, Boqia Xie

**Affiliations:** 1 Department of Cardiology, Cardiovascular Imaging Center, Beijing Chaoyang Hospital, Capital Medical University, Beijing 100020, China; 2 School of Computer Science, Chengdu University of Information Technology, Chengdu 610225, China; 3 Hopkins School, New Haven, CT 06515, USA

**Keywords:** acute myocardial infarction, coronary artery disease, archaeome, gut microbiome, shotgun metagenomics

## Abstract

Cardiovascular microbiome research has focused mainly on bacterial taxa and pathways. We profiled stool archaea, bacteria, fungi, and viruses in patients with acute myocardial infarction (AMI) and healthy controls. Paired plasma metabolomics was examined in a subset. An independent angiography-defined cohort included angiographically normal controls, severe coronary artery disease (CAD), and AMI. No archaeal genus remained differentially abundant after multiple-testing correction. In the discovery cohort, archaeal-bacterial correlations were predominantly positive in healthy controls and negative in AMI, while archaeal-fungal rewiring was prominent. The extension cohort identified sign-flip archaeal-virome edges between severe CAD and AMI, while severe CAD showed the lowest archaeal-bacterial connectivity. Plasma metabolomics captured a broad AMI-associated systemic shift. These findings show that gut archaeal signals are expressed through multi-kingdom ecological organization across coronary disease states.

## Highlights

• Archaeal-bacterial correlations differ in direction between healthy controls and AMI
• Archaeal-fungal rewiring dominates the discovery cohort AMI contrast
• Severe CAD shows the lowest archaeal-bacterial connectivity
• Archaeal-virome sign flips characterize the severe CAD-AMI contrast

## Graphical abstract

**Figure:**
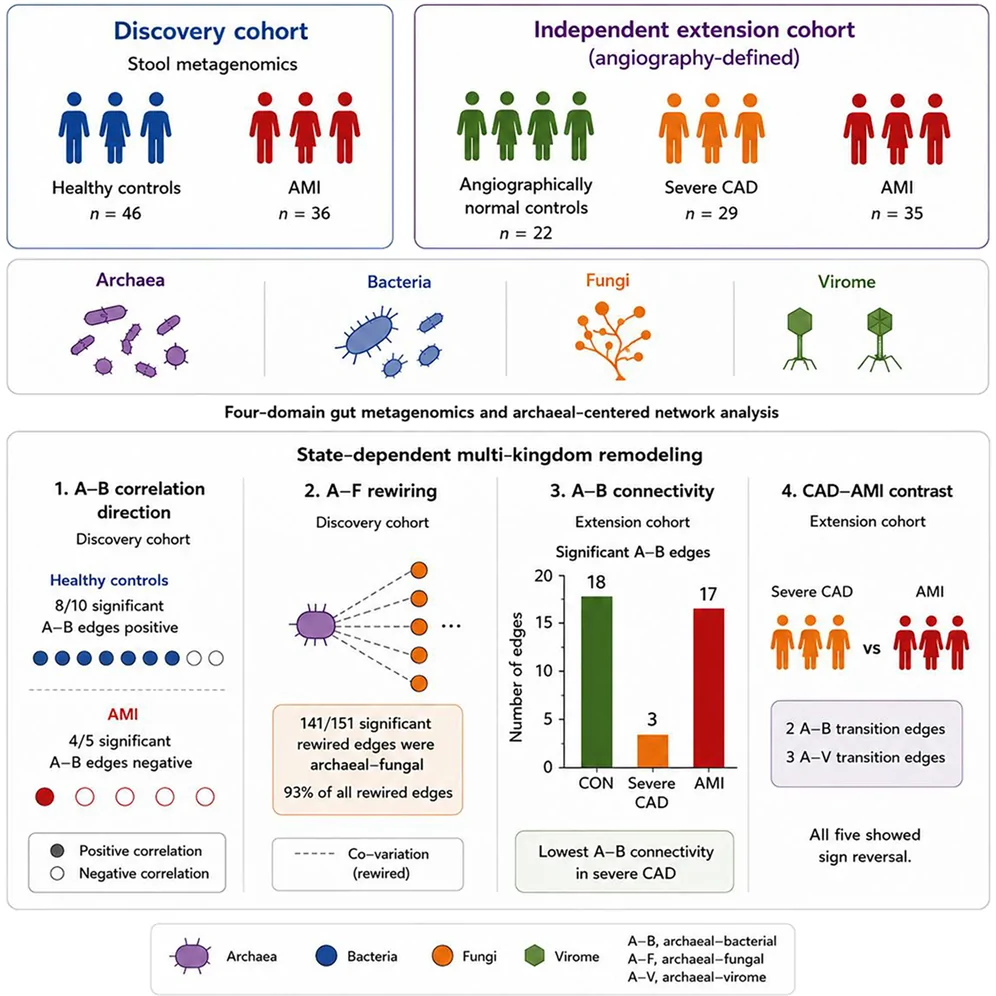

## Teaser

Health sciences; Medicine; Medical specialty; Internal medicine; Cardiovascular medicine

## Introduction

Coronary artery disease (CAD) and acute myocardial infarction (AMI) continue to drive substantial morbidity and mortality worldwide. A growing body of human metagenomic studies and mechanistic experiments has implicated the gut microbiome in atherosclerosis and thrombotic risk, expanding cardiovascular pathophysiology beyond traditional host-centric models,^1^^,^^2^^,^^3^^,^^4^^,^^5^^,^^6^ yet most cardiovascular microbiome investigations still focus predominantly on bacterial taxa and bacterial pathways.^7^

A major blind spot is the archaeome. Archaea are increasingly recognized as core residents of the human gastrointestinal tract, and methanogenic lineages such as *Methanobrevibacter, Methanosphaera, Methanosarcina* and members of *Methanomassiliicoccales* are prevalent and metabolically active.^8^^,^^9^^,^^10^^,^^11^^,^^12^^,^^13^^,^^14^^,^^15^^,^^16^^,^^17^ By consuming H_2_/CO_2_ and, in some lineages, methylated amines, and through interspecies hydrogen transfer, methanogens can reshape fermentation ecology and luminal redox conditions—features that plausibly intersect with cardiometabolic microbial functions.^9^^,^^10^^,^^11^^,^^14^^,^^15^^,^^16^^,^^17^ Yet archaeal community structure and functional potential remain underprofiled in AMI cohorts, and their embedding within a multi-domain gut ecosystem is largely undefined. This gap is particularly relevant given that fungi, microbial eukaryotes and viruses—can restructure bacterial consortia and host immune tone and are altered across multiple inflammatory and metabolic conditions.^18^^,^^19^^,^^20^^,^^21^^,^^22^^,^^23^^,^^24^^,^^25^^,^^26^ Moreover, interaction-like structure inferred from sequencing data can be confounded by compositionality and indirect associations, motivating cross-kingdom analyses that explicitly account for these constraints.^27^^,^^28^^,^^29^^,^^30^

To address these gaps, we performed an archaeal-centered, four-domain (archaea-bacteria-fungi-viruses) stool metagenomic study in AMI and healthy controls, using false-discovery rate (FDR)-controlled cross-domain correlation screening and differential-correlation (rewiring) analyses to delineate how gut archaea are embedded within multi-kingdom community structure. We prioritized methanogenic taxa and prespecified archaeal functional regions of interest (ROIs) to link archaeal ecological configurations to cardiometabolic-relevant metabolic niches. Finally, we analyzed an independent extension cohort spanning angiographically normal controls, angiographically severe CAD and AMI to separate severity-linked patterns from CAD-AMI transition signals in archaeal-anchored cross-domain networks, thereby clarifying whether observed AMI-healthy-control differences reflect chronic atherosclerotic burden or acute event-state remodeling.

## Results

### Analytic sets

**Figure 1:**
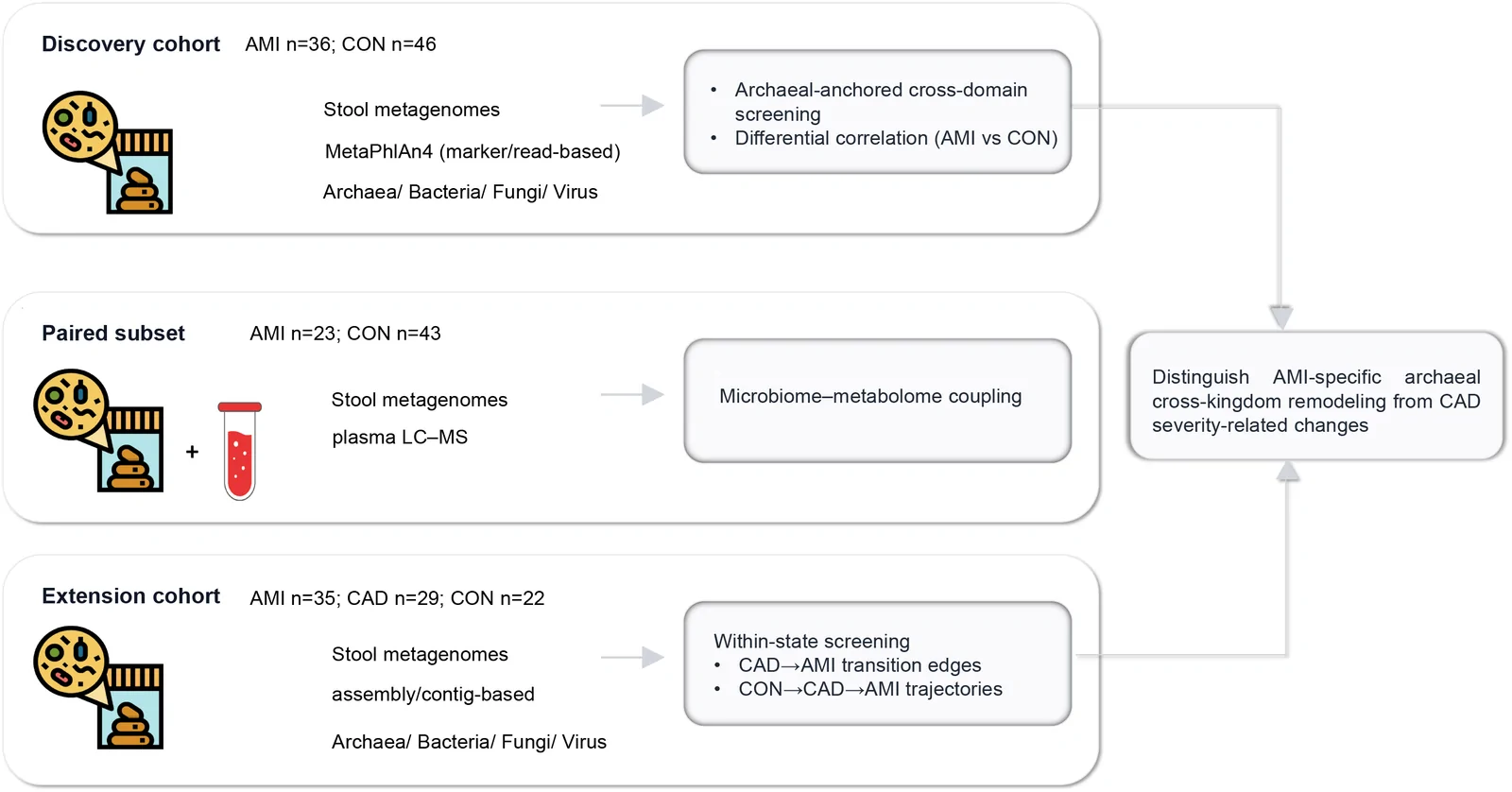
Study design and prespecified analytic workflow Overview of the two-stage archaeal-centered study design. In the discovery cohort, stool shotgun metagenomes from patients with acute myocardial infarction (AMI) and healthy controls were profiled at the genus level across four microbial domains: archaea, bacteria, fungi, and viruses. This cohort was used for prespecified archaeal-centered cross-domain screening, AMI-healthy-control differential-correlation analysis, and paired stool-plasma microbiome-metabolome coupling. The extension cohort of angiographically normal controls (CON), severe coronary artery disease (CAD), and AMI was used for prespecified within-state screening, CAD-AMI transition testing, and three-state trajectory summaries.

The study design and prespecified analytic workflow are shown in Figure 1. Participant characteristics of the discovery cohort (AMI: *n* = 36; healthy controls: *n* = 46) and the angiography-defined extension cohort (CON: *n* = 22; CAD: *n* = 29; AMI: *n* = 35) were summarized in Table 1. Participants were recruited across three campuses of Beijing Chaoyang Hospital under harmonized sample collection and storage procedures. Across cohorts, age and BMI were broadly comparable across groups, whereas sex distribution and several cardiometabolic or clinical variables differed as expected for AMI and severe CAD.

**Table 1:** Baseline characteristics for the discovery cohort and extension cohort

Variables	Discovery cohort			Extension cohort			
	AMI (*N* = 36)	Healthy controls (*N* = 46)	*p* Value	AMI (*N* = 35)	CAD (*N* = 29)	CON (*N* = 22)	*p* Value
**Clinical covariates used for sensitivity analyses**							

Female, *N* (%)	6 (16.67%)	18 (39.13%)	0.03	7 (20.00%)	13 (44.83%)	17 (77.27%)	<0.001
Age, years	57.94 ± 11.55	58.98 ± 10.33	0.67	60.46 ± 12.93	64.17 ± 9.22	61.41 ± 9.83	0.40
Body mass index, kg/m^2^	24.89 (23.80, 28.57)	24.97 (23.32, 27.59)	0.47	24.33 (22.03, 29.36)	25.10 (22.77, 28.34)	26.13 (23.58, 27.56)	0.79
Smoking, *N* (%)	18 (50.00%)	11 (23.91%)	0.01	18 (51.43%)	12 (41.38%)	5 (22.73%)	0.01
Drinking, *N* (%)	11 (30.56%)	9 (19.57%)	0.25	15 (42.86%)	6 (20.69%)	1 (4.55%)	<0.01
Hypertension, *N* (%)	15 (41.67%)	19 (41.30%)	0.97	21 (60.00%)	24 (82.76%)	15 (68.18%)	0.14
Diabetes mellitus, *N* (%)	10 (27.78%)	14 (30.43%)	0.79	15 (42.86%)	5 (17.24%)	4 (18.18%)	0.04
Hyperlipidemia, *N* (%)	36 (100.00%)	21 (45.65%)	<0.001	28 (80.00%)	16 (55.17%)	8 (36.36%)	<0.01

**Cardiac and laboratory characteristics**							

Left ventricular end-diastolic diameter, mm	48.00 (45.25, 50.75)	48.00 (45.00, 51.00)	0.82	47.00 (45.00, 50.00)	46.00 (44.00, 49.00)	48.00 (44.00, 49.00)	0.63
Left ventricular end-systolic diameter, mm	32.00 (29.25, 35.75)	31.00 (27.00, 33.00)	0.14	31.00 (27.00, 35.00)	29.00 (27.55, 33.25)	30.00 (27.00, 31.00)	0.65
Left ventricular ejection fraction, %	59.50 (48.50, 63.00)	65.00 (64.00, 71.00)	<0.001	68.00 (60.40, 71.85)	67.00 (64.00, 69.00)	68.00 (65.50, 70.50)	0.58
White blood cell, 10^9^/L	9.75 (7.95, 12.68)	6.31 (5.38, 7.43)	<0.001	9.30 (8.10, 12.70)	6.30 (5.40, 7.10)	7.10 (5.50, 7.70)	<0.001
Lactate dehydrogenase, U/L	433.50 (248.08, 773.25)	161.00 (148.00, 183.00)	<0.001	287.80 (216.08, 565.68)	175.90 (156.60, 193.53)	178.60 (141.00, 203.60)	<0.001
Cardiac Troponin I maximum, ng/mL	45.21 (14.43, 100.00)	–	–	11.20 (3.15, 34.67)	–	–	–
Creatine kinase-MB, U/L	30.19 (17.15, 79.58)	–	–	10.23 (3.18, 36.42)	–	–	–
NT-Brain natriuretic peptide, pg/mL	807.75 (300.93, 1474.28)	–	–	599.55 (287.60, 938.98)	–	–	–
Uric acid, umol/L	360.89 ± 106.94	333.87 ± 78.74	0.30	341.60 (284.20, 392.55)	301.70 (271.60, 345.60)	308.60 (263.00, 361.10)	0.31
Fasting glucose, mmol/L	7.10 (5.72, 9.08)	4.77 (4.06, 5.92)	<0.001	6.85 (6.21, 8.91)	5.50 (5.08, 7.25)	5.58 (4.61, 6.13)	<0.001
Hemoglobin A1c, %	6.00 (5.70, 6.55)	6.75 (6.03, 7.20)	0.02	6.10 (5.70, 7.70)	6.00 (5.70, 6.75)	6.20 (5.75, 6.60)	0.49
Blood urea nitrogen, mmol/L	5.23 (4.27, 6.60)	2.90 (2.45, 3.39)	<0.001	5.84 ± 1.87	5.68 ± 1.24	6.08 ± 1.52	0.70
Creatinine, μmol/L	69.65 (57.35, 83.20)	68.00 (64.20, 80.90)	0.99	73.65 (64.50, 85.08)	69.90 (62.40, 78.00)	64.60 (57.50, 73.00)	0.03
Total cholesterol, mmol/L	4.51 (4.13, 5.67)	3.55 (3.25, 4.53)	0.001	4.36 ± 1.05	4.46 ± 0.97	4.23 ± 0.78	0.70
Triglyceride, mmol/L	1.48 (1.17, 2.20)	1.16 (0.86, 1.61)	0.04	1.53 (1.14, 2.75)	1.76 (1.14, 2.25)	1.49 (0.90, 2.19)	0.94
High-density lipoprotein cholesterol, mmol/L	0.98 (0.90, 1.23)	1.00 (0.81, 1.19)	0.51	1.18 ± 0.36	1.27 ± 0.27	1.20 ± 0.27	0.48
Low-density lipoprotein cholesterol, mmol/L	3.60 ± 1.22	2.21 ± 0.77	<0.001	2.86 ± 0.95	2.92 ± 1.34	2.50 ± 0.60	0.33
Lipoprotein(a), mg/L	31.10 (8.90, 80.30)	19.05 (9.20, 27.25)	0.13	69.90 (34.75, 144.83)	143.50 (72.55, 445.68)	63.60 (39.48, 289.88)	0.12
D-dimer, mg/L	0.21 (0.14, 0.62)	0.25 (0.17, 0.36)	0.77	0.09 (0.06, 0.14)	0.11 (0.06, 0.17)	0.06 (0.04, 0.08)	0.08

AMI, acute myocardial infarction; CAD, angiographically severe coronary artery disease; CON, angiographically normal controls in the extension cohort; IQR, interquartile range; SD, standard deviation; “–” indicates data not available.

The variables listed under “Clinical covariates used for sensitivity analyses” were included in covariate-aware residualization analyses. Continuous variables shown as mean ± SD were compared using Student’s *t* test or one-way analysis of variance (ANOVA); continuous variables shown as median (IQR) were compared using the Wilcoxon rank-sum test or Kruskal-Wallis test. Categorical variables are shown as *N* (%) and were compared using the χ^2^ test or Fisher’s exact test, as appropriate. *p* values are descriptive and unadjusted.

**Figure 2:**
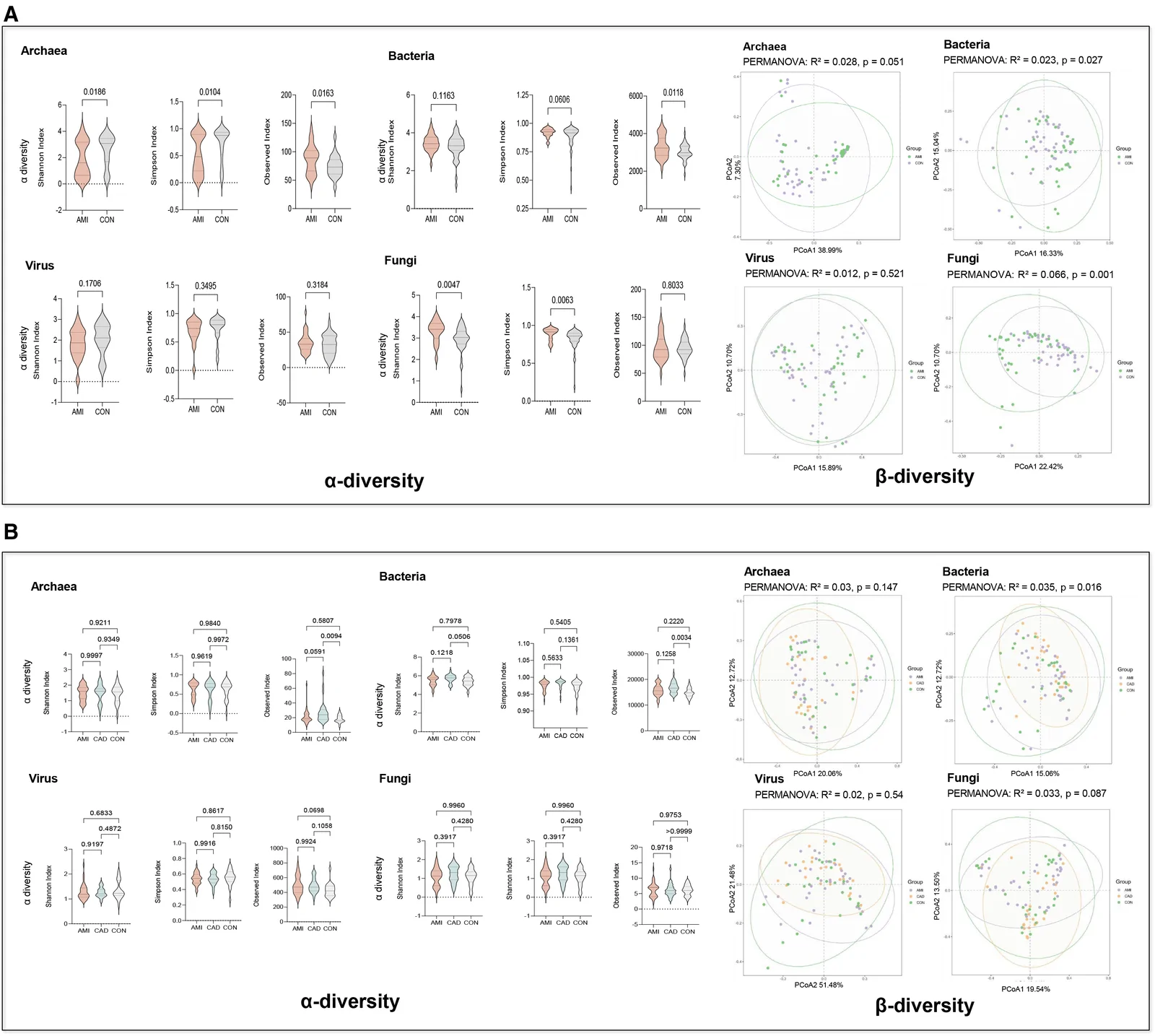
Descriptive domain-specific α- and β-diversity analyses (A) Discovery cohort: descriptive α-diversity and β-diversity analyses across the archaeome, bacteriome, mycobiome, and virome in AMI and healthy controls. (B) Extension cohort: corresponding descriptive diversity analyses across CON, CAD, and AMI. PCoA, principal coordinates analysis.

Domain-specific α- and β-diversity profiles of the archaeome, bacteriome, mycobiome, and virome are shown in Figure 2. At the archaeal genus level, no individual genus remained differentially abundant after multiple-testing correction in either the discovery AMI-healthy-control comparison or the extension CON-CAD-AMI comparison.

### Genus-level archaeal-centered correlation screening indicates an AMI-associated A-B sign shift and expanded archaeal-virome co-variation

**Figure 3:**
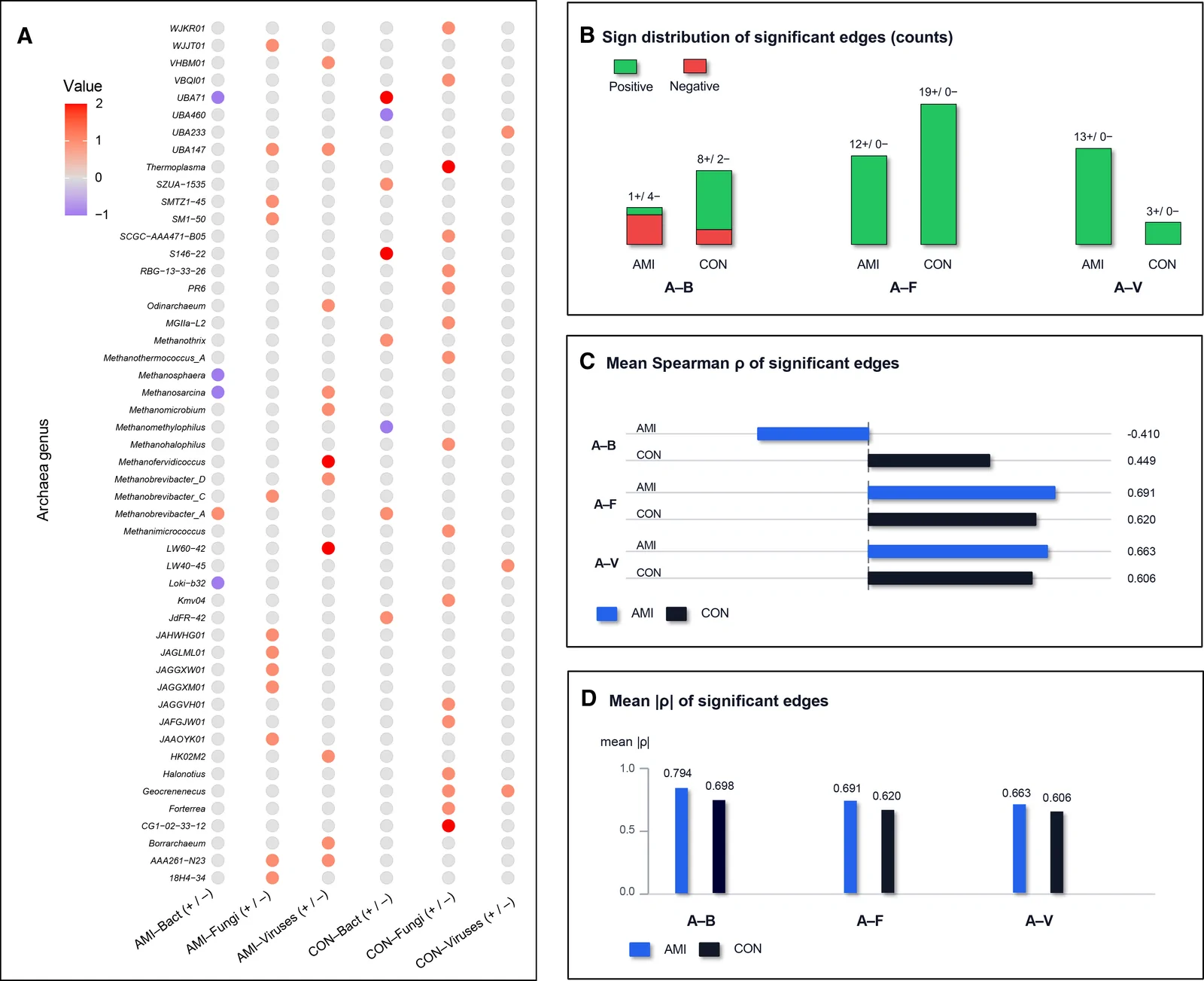
Prespecified archaeal-centered cross-domain correlation screening (A) Archaeal genera involved in at least one FDR-significant archaeal-bacterial (A-B), archaeal-fungal (A-F), or archaeal-viral (A-V) co-variation edge in AMI or healthy controls; dot color denotes Spearman ρ on CLR-transformed abundances. (B) Edge counts by domain pair and sign in AMI and healthy controls. (C) Mean absolute correlation strength across significant edges. FDR, false-discovery rate; CLR, centered log-ratio.

We screened archaeal-centered cross-domain associations by Spearman correlations on centered log-ratio (CLR)-transformed genus abundances within AMI and healthy controls, controlling multiplicity within each group × domain-pair family (BH-FDR q ≤ 0.10; Figure 3; Table 2). Significant edges were sparse but domain-structured. In the archaeal-bacterial (A-B) layer, AMI correlations were predominantly negative (4/5 edges), whereas healthy-control A-B correlations were predominantly positive (8/10 edges).

**Table 2:** Directional distribution of archaeal cross-domain correlations in the discovery cohort

Group	Domain	Archaea genera (*q* ≤ 0.10)	Edges (q ≤ 0.10)	Positive edges	Negative edges	Mean ρ	Mean |ρ|
AMI	Bacteria	5	5	1	4	−0.410	0.794
AMI	Fungi	12	12	12	0	0.691	0.691
AMI	Viruses	11	13	13	0	0.663	0.663
Healthy controls	Bacteria	8	10	8	2	0.449	0.698
Healthy controls	Fungi	17	19	19	0	0.620	0.620
Healthy controls	Viruses	3	3	3	0	0.606	0.606

AMI, acute myocardial infarction.

In contrast, all significant archaeal-fungal (A-F) and archaeal-viral (A-V) co-variation edges were positive in both groups. The A-V layer was denser in AMI than in healthy controls (13 edges involving 11 archaeal genera vs. 3 edges involving 3 archaeal genera), while A-F associations were present in both groups but were driven by largely non-overlapping archaeal taxa.

At the taxon level, *Methanobrevibacter_A* retained a positive A-B association in both groups. AMI-specific negative A-B edges involved *Methanosarcina, Methanosphaera*, and the *Asgard lineage Loki-b32*, whereas healthy controls showed a positive A-B edge for the acetoclastic methanogen *Methanothrix* and a negative A-B edge for the methylamine-utilizing methanogen *Methanomethylophilus*. AMI-linked A-V signals included *Methanofervidicoccus* and *Odinarchaeum*, whereas healthy-control-associated A-F signals included MGIIa-L2 and *Thermoplasma* (Figure 3A).

### Functional archaeal ROIs nominate an annotation-derived trimethylamine *N*-oxide-linked electron-transfer score as the broadest multi-kingdom correlate

We correlated three prespecified stool MetaCyc-derived archaeal functional ROI scores with screened bacterial (top-variance 300), fungal, and viral genera within AMI and healthy controls (Table 3). Methanogenesis-linked scores showed limited signal at genus resolution: methanogenesis_H2_score yielded no significant associations with bacteria or viruses in either group and only one positive association with fungi in AMI, while methanogenesis_acetate_score showed two bacterial associations per group with mixed direction and no fungal or viral hits. In contrast, trimethylamine *N*-oxide (TMAO)_ET_score showed the most extensive and directionally consistent coupling, with 14 positive bacterial associations in AMI and 12 in controls, together with additional positive links to a small number of fungal and viral genera. Collectively, these results indicate that, under FDR control, the annotation-derived TMAO_ET score was the broadest stool archaeal functional ROI correlate of multi-kingdom community variation, whereas methanogenesis scores were comparatively weakly connected at genus resolution.

**Table 3:** Cross-domain associations for stool MetaCyc-derived archaeal functional ROI scores

ROI	Group	Domain	Tested genera (pairs)	Significant edges	Positive edges	Negative edges
**Methanogenesis_H2_score**	AMI	Bacteria	300	0	0	0
	Healthy controls	Bacteria	300	0	0	0
	AMI	Fungi	224	1	1	0
	Healthy controls	Fungi	223	0	0	0
	AMI	Viruses	84	0	0	0
	Healthy controls	Viruses	80	0	0	0
**Methanogenesis_acetate_score**	AMI	Bacteria	300	2	1	1
	Healthy controls	Bacteria	300	2	1	1
	AMI	Fungi	224	0	0	0
	Healthy controls	Fungi	223	0	0	0
	AMI	Viruses	84	0	0	0
	Healthy controls	Viruses	80	0	0	0
**TMAO_ET_score**	AMI	Bacteria	300	14	14	0
	Healthy controls	Bacteria	300	12	12	0
	AMI	Fungi	224	1	1	0
	Healthy controls	Fungi	223	2	2	0
	AMI	Viruses	84	4	4	0
	Healthy controls	Viruses	80	1	1	0

AMI, acute myocardial infarction; ROI, region of interest; TMAO, trimethylamine *N*-oxide.

### Conditional (module-level) networks remain FDR-sparse under permutation-controlled inference

**Figure 4:**
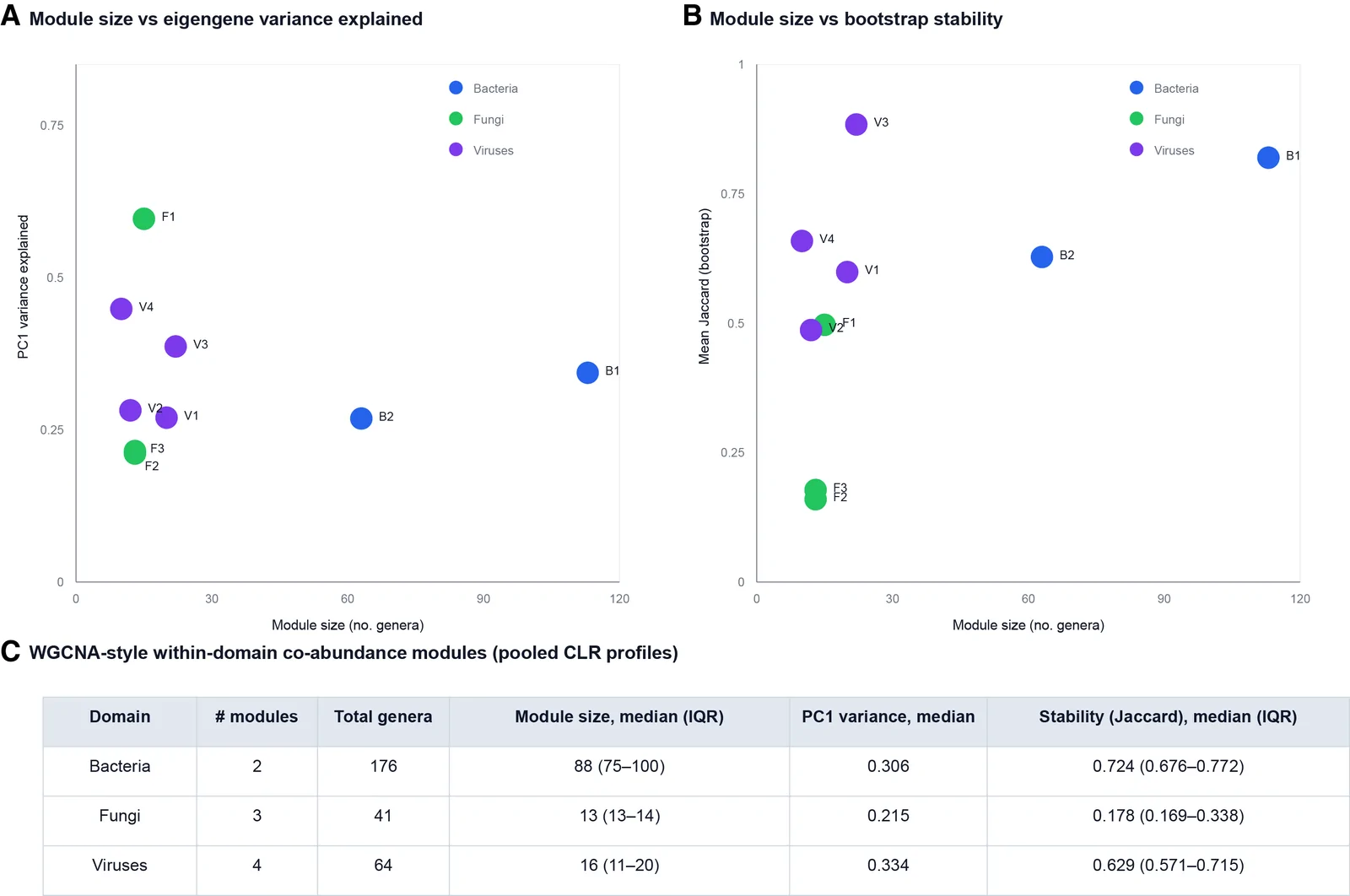
Prespecified module construction and descriptive stability assessment (A) Module size versus eigengene variance explained by the first principal component (PC1). (B) Module size versus bootstrap stability, quantified by mean Jaccard similarity across resamples. (C) Summary of module number, represented genera, module size, eigengene variance explained, and stability metrics. Module construction was prespecified; stability metrics were descriptive. B, F, and V denote bacterial, fungal, and viral modules, respectively; numeric suffixes indicate within-domain module identifiers only. Detailed module composition, eigengene variance explained, stability metrics, and member genera are provided in Table S1.

After summarizing bacteria, fungi, and viruses into within-domain co-abundance modules, module properties and bootstrap stability metrics are summarized in Figure 4 and Table S1. We then estimated archaeal-anchored near-partial correlations between archaeal genera and partner-domain module eigengenes. No archaeal-module edge remained significant after permutation-controlled inference in either AMI or healthy controls, indicating that the genus-level cross-domain co-abundance patterns are largely explained by shared embedding in broader community structure rather than by strong direct archaeal-partner dependencies detectable under conditional modeling.

### Differential correlation testing localizes AMI-healthy-control rewiring predominantly to the archaeal-fungal layer

**Figure 5:**
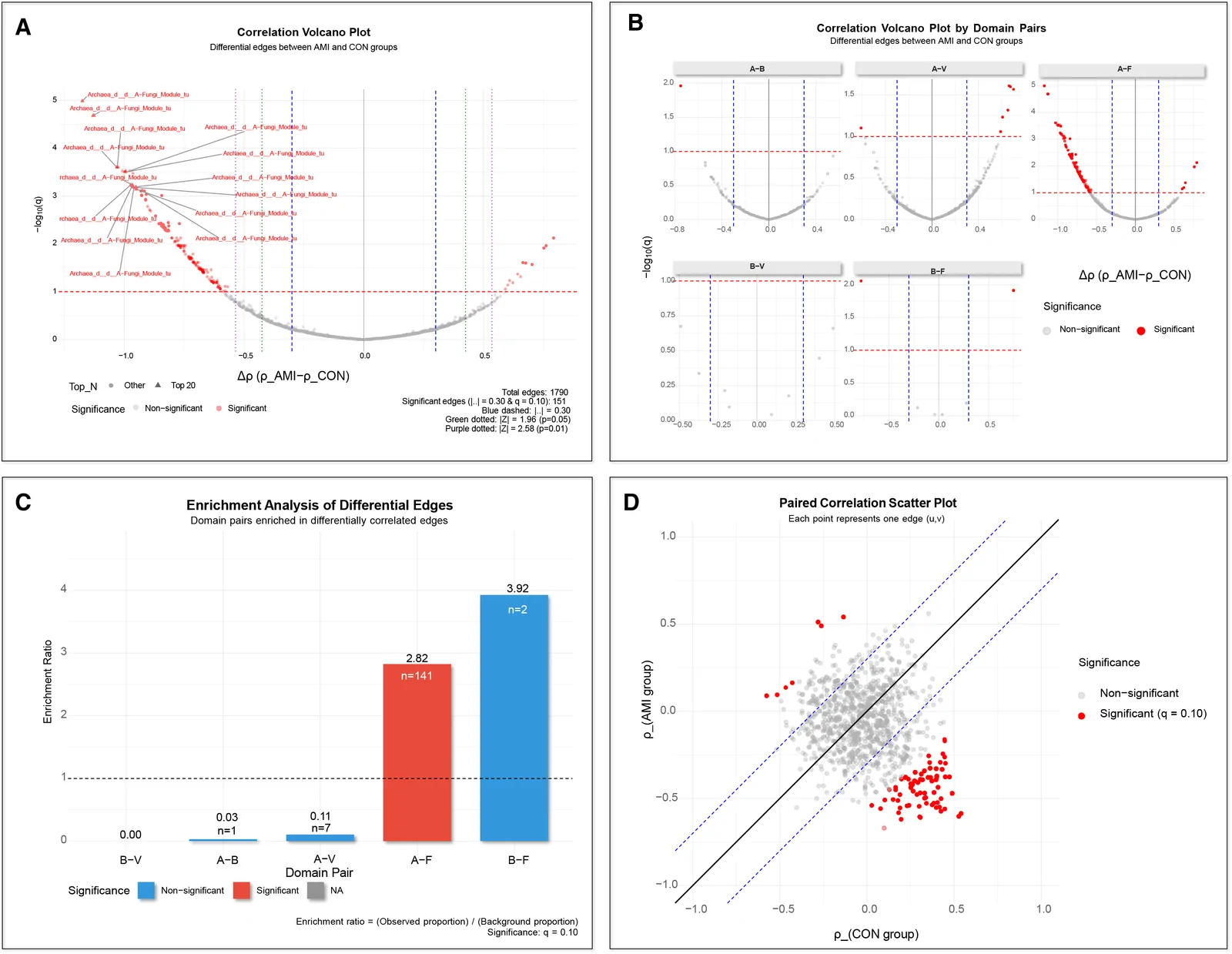
Prespecified AMI-healthy-control differential-correlation analysis (A) Volcano plot of differential correlations across tested cross-domain edges; Δρ = ρ_AMI − ρ_control, where “control” denotes healthy controls. (B) Domain-pair-stratified volcano plots. (C) Enrichment of rewired edges by domain pair relative to all tested edges. (D) Group-specific correlations for all tested edges. Rewired edges were defined as |Δρ| ≥ 0.30 and BH-FDR q_Δ ≤ 0.10. BH-FDR, Benjamini-Hochberg FDR.

Across 1,790 cross-domain edges tested, 151 (8.4%) met the prespecified criteria for differential correlation (|Δρ|≥0.30; BH-FDR q ≤ 0.10; Figure 5A), with many edges showing attenuation or sign reversal in AMI relative to healthy controls (Figure 5D). Rewiring was concentrated in the A-F layer: 141/151 (93%) significant edges were A-F, whereas 7 were A-V and 1 was A-B (with 2 additional B-F edges; Figures 5A–5C). Thus, AMI-associated cross-domain remodeling was detectable at the marginal-correlation level, but was overwhelmingly concentrated in the A-F layer.

### Extension cohort supports a CAD-associated A-B low-connectivity state and a virome-associated CAD-AMI transition pattern

In the extension cohort (CON: *n* = 22; CAD: *n* = 29; AMI: *n* = 35), the screened node set comprised 31 archaeal genera, 300 variance-ranked bacterial genera, 38 viral genera, and 9 fungal genera.

**Figure 6:**
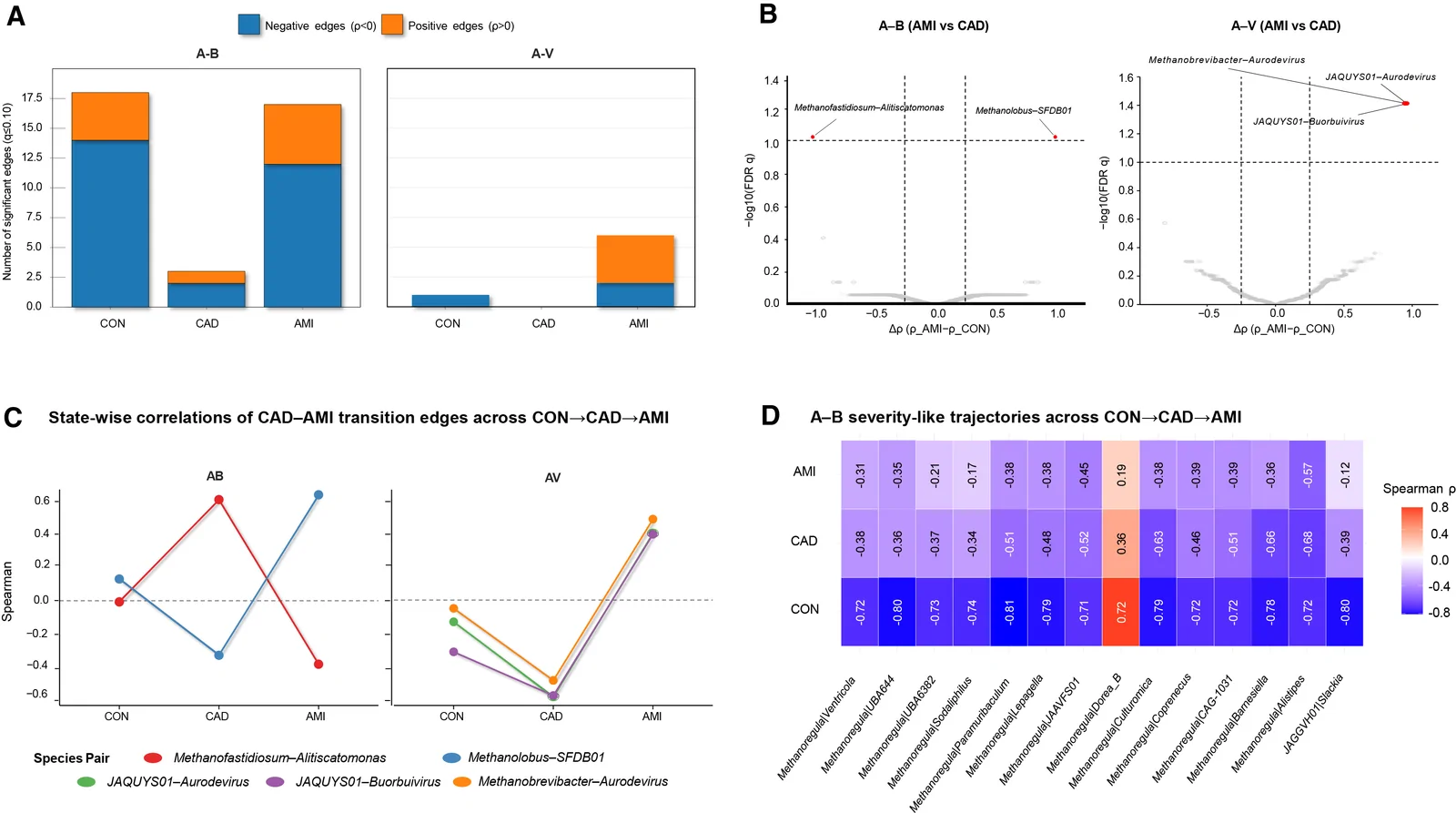
Prespecified extension-cohort CON-CAD-AMI analysis (A) Within-state archaeal-centered screening in CON, CAD, and AMI. (B) CAD-AMI differential-correlation testing for A-B and A-V edges. (C) State-wise correlations for CAD-AMI transition edges. (D) Descriptive heatmap of severity-like A-B trajectories, defined as monotonic change in |ρ| across CON-CAD-AMI without sign reversal; color encodes Spearman ρ. CON denotes angiographically normal controls; A-B, archaeal-bacterial; A-V, archaeal-viral co-variation.

In within-group archaeal-centered correlation screening (Figure 6A; Table S2), A-B connectivity reached a nadir in severe CAD (3 significant edges) compared with CON (18 edges) and AMI (17 edges). A-B edges were predominantly negative in all three states (CON: 14/18; CAD: 2/3; AMI: 12/17). As a descriptive degree summary, CON A-B edges were concentrated on *Methanoregula* (14/18), whereas AMI A-B edges were concentrated on HRBIN01 (11/17); CAD showed only sparse A-B connectivity.

Archaeal-viral (A-V) co-variation was observed primarily in AMI (6 edges in AMI vs. 1 in CON and 0 in CAD). No A-F edge reached q ≤ 0.10, consistent with the small fungal feature set retained under prespecified thresholds (9 genera).

Among A-B edges significant in at least one state, 14 exhibited monotonic changes in correlation strength |ρ| across CON-CAD-AMI without sign reversal (“severity-like” trajectories; Figure 6D; Table S3), dominated by *Methanoregula* (12/14).

Direct CAD versus AMI differential correlation testing identified five CAD-AMI transition edges at q ≤ 0.10 (2 A-B and 3 A-V; Figures 6B and 6C; Table 4). All five transition edges showed sign reversal between CAD and AMI, with the strongest signals in the A-V layer (Table 4).

**Table 4:** AMI-specific CAD→AMI transition edges identified by differential correlation testing

Domain pair	Archaeal genus	Partner genus	ρ_CON	ρ_CAD	ρ_AMI	Δρ (AMI−CAD)	FDR q_diff
A-B	*Methanofastidiosum*	*Alitiscatomonas*	−0.005	0.622	−0.393	−1.015	0.0935
A-B	*Methanolobus*	*SFDB01*	0.132	−0.340	0.648	0.988	0.0935
A-V	*Methanobrevibacter*	*Aurodevirus*	−0.049	−0.472	0.475	0.946	0.0385
A-V	*JAQUYS01*	*Aurodevirus*	−0.128	−0.567	0.392	0.958	0.0385
A-V	*JAQUYS01*	*Buorbuivirus*	−0.304	−0.561	0.386	0.947	0.0385

AMI, acute myocardial infarction; CAD, angiographically severe coronary artery disease; CON, angiographically normal controls in the extension cohort; FDR, false discovery rate; A-B, archaeal-bacterial; A-V, archaeal-viral.

ROI scores themselves did not differ across CON, CAD, and AMI, and under the prespecified primary differential-correlation framework, no ROI-bacterial or ROI-viral edge reached qdiff≤0.10 (Table S4). Although within-state ROI correlations were detected, these signals did not translate into CAD-AMI transition edges under the primary differential-correlation analysis.

Covariate-adjusted sensitivity analyses are summarized in Table 5. Discovery AMI A-F and A-V edge sets retained their direction and q ≤ 0.10 significance after adjustment for measured clinical covariates, while extension CAD-AMI transition-Δ directions remained stable despite attenuation of FDR significance for several extension within-state networks. Bootstrap/permutation analyses of headline discovery rewired edges and extension CAD-AMI transition edges showed stable Δρ directions, with percentile 95% confidence intervals (CIs)excluding zero and empirical permutation q ≤ 0.10 across tested edge sets (Table S5). Table 6 summarizes the evidence hierarchy and cross-cohort comparability of the major archaeal-centered findings.

**Table 5:** Covariate-adjusted sensitivity analyses for archaeal-centered network findings

Cohort	Analysis	Network layer	Sample set/contrast	Complete-case n	Edges/pattern evaluated	Preserved direction/pattern	q ≤ 0.10 after adjustment	Effect-size summary
**Discovery**	Edge stability	A-F	AMI	36	12 AMI significant edges	12/12	12/12	Median |ρ| 0.662 → median |adjusted r| 0.607
	Edge-set retest	A-F	Healthy controls	42	12 AMI-derived edges	7/12	0/12	Median |adjusted r| 0.100
	Edge stability	A-V	AMI	36	13 AMI significant edges	13/13	13/13	Median |ρ| 0.645 → median |adjusted r| 0.631
	Edge-set retest	A-V	Healthy controls	42	13 AMI-derived edges	4/13	1/13	Median |adjusted r| 0.115
	Sign-composition stability	A-B	AMI	36	All candidate A-B edges	Positive: 57.2%→58.0%; negative: 42.8%→42.0%	Not edge-selected	Median |ρ| 0.123 → median |adjusted r| 0.127
	Sign-composition stability	A-B	Healthy controls	42	All candidate A-B edges	Positive: 54.8%→55.0%; negative: 45.2%→45.0%	Not edge-selected	Median |ρ| 0.116 → median |adjusted r| 0.118
**Extension**	Edge stability	A-B	CON	19	18 significant within-state edges	18/18	0/18	Median |ρ| 0.739 → median |adjusted r| 0.826
	Edge stability	A-B	CAD	27	3 significant within-state edges	3/3	0/3	Median |ρ| 0.714 → median |adjusted r| 0.615
	Edge stability	A-B	AMI	35	17 significant within-state edges	17/17	5/17	Median |ρ| 0.627 → median |adjusted r| 0.628
	Edge stability	A-V	CON	19	1 significant within-state edge	1/1	0/1	Median |ρ| 0.772 → median |adjusted r| 0.815
	Edge stability	A-V	AMI	35	6 significant within-state edges	6/6	0/6	Median |ρ| 0.587 → median |adjusted r| 0.559
**Extension**	Transition-Δ stability	A-B	CAD vs. AMI	CON 19/CAD 27/AMI 35	2 significant differential edges	AMI–CAD Δ: 2/2	Not assessed for differential q	AMI–CON Δ: 2/2; CAD–CON Δ: 2/2
	Transition-Δ stability	A-V	CAD vs. AMI	CON 19/CAD 27/AMI 35	3 significant differential edges	AMI–CAD Δ: 3/3	Not assessed for differential q	AMI–CON Δ: 3/3; CAD–CON Δ: 2/3

AMI, acute myocardial infarction; CAD, angiographically severe coronary artery disease; CON, angiographically normal controls in the extension cohort; A-B, archaeal-bacterial; A-F, archaeal-fungal; A-V, archaeal-viral.

A-B, A-F, and A-V denote archaeal-bacterial, archaeal-fungal, and archaeal-viral co-variation layers, respectively. Genus-level CLR profiles were rank-residualized against age, sex, BMI, smoking, drinking, diabetes, hyperlipidemia, and hypertension where estimable. Complete-case sample sizes are shown. “Preserved direction/pattern” indicates that the adjusted residual correlation, sign-composition pattern, or between-state Δ retained the same direction as the corresponding unadjusted result. q values were calculated using Benjamini-Hochberg FDR correction within each edge family. Transition-Δ rows summarize adjusted direction preservation rather than adjusted differential-edge significance. No significant within-state A-F edges and no significant CAD within-state A-V edges were identified in the extension cohort. Residual and unmeasured confounding remains possible.

**Table 6:** Evidence hierarchy and cross-cohort comparability of major archaeal-centered findings

Finding	Discovery cohort	Extension cohort	Evidence category	Interpretation level
Network-centered archaeal signal	Archaeal-centered cross-domain correlations formed the main signal; robust archaeal differential abundance was not the primary finding.	Analyses emphasized within-state and transition-network structure rather than pooled taxonomic abundance.	Primary framework finding	Network-level archaeal signal
A-B state organization	AMI and healthy controls showed distinct A-B sign structure.	CAD showed low A-B connectivity, whereas AMI showed renewed A-B connectivity with different leading archaeal partners.	Primary network finding	Domain-layer ecological organization
A-F rewiring	A-F layer contributed prominently to AMI-healthy-control differential rewiring.	Fungal feature retention was limited; no significant within-state A-F edges were detected.	Discovery-dominant finding	Requires deeper mycobiome validation
A-V co-variation	A-V co-variation was enriched in AMI relative to healthy controls.	A-V edges contributed to CAD-AMI transition patterns.	Primary transition-related finding	Virome-associated co-variation pattern
Severe CAD low-connectivity state	Not assessed in the binary AMI-healthy-control design.	CAD showed the lowest A-B connectivity among CON, CAD, and AMI.	Extension-specific finding	Three-state disease-context signal
CAD-AMI transition edges	Not assessed in the binary AMI-healthy-control design.	A-B and A-V differential edges distinguished CAD from AMI.	Extension-specific transition finding	CAD-AMI transition-Δ pattern
TMAO_ET functional ROI coupling	Annotation-derived TMAO_ET score showed broader cross-domain coupling than methanogenesis-linked scores.	ROI scores did not define CAD-AMI transition edges in the primary extension analysis.	Supportive functional annotation signal	Ecological ROI, not biochemical flux readout
Plasma metabolome separation	Plasma LC-MS showed broad AMI-healthy-control differential features.	Not assessed in the extension cohort.	Supportive systemic-metabolic signal	Circulating AMI-associated metabolome shift
Archaeal ROI–metabolite coupling	No archaeal ROI-metabolite association survived BH-FDR correction.	Not assessed in the extension cohort.	Negative boundary condition	No FDR-robust stool-plasma metabolite bridge
AMI severity anchoring	Candidate archaeal taxa were associated with peak cTnI and LVEF within AMI.	Selected associations were evaluated in the extension AMI set and synthesized across cohorts.	Exploratory clinical anchoring	Candidate clinical association

AMI, acute myocardial infarction; CAD, angiographically severe coronary artery disease; CON, angiographically normal controls in the extension cohort; A-B, archaeal-bacterial; A-F, archaeal-fungal; A-V, archaeal-viral; FDR, false discovery rate; BH-FDR, Benjamini-Hochberg false discovery rate; TMAO, trimethylamine *N*-oxide; ROI, region of interest; LC-MS, liquid chromatography-mass spectrometry; cTnI, cardiac troponin I; LVEF, left ventricular ejection fraction.

A-B, A-F, and A-V denote archaeal-bacterial, archaeal-fungal, and archaeal-viral co-variation layers, respectively. TMAO_ET denotes the annotation-derived TMAO-linked electron-transfer ROI. The discovery cohort used marker-based read profiling, whereas the extension cohort used an assembly/contig-based strategy. Evidence categories indicate the inferential role of each finding in the revised manuscript; cross-cohort entries reflect pattern-level comparability rather than direct feature-level replication.

### Microbiome-metabolome coupling

**Figure 7:**
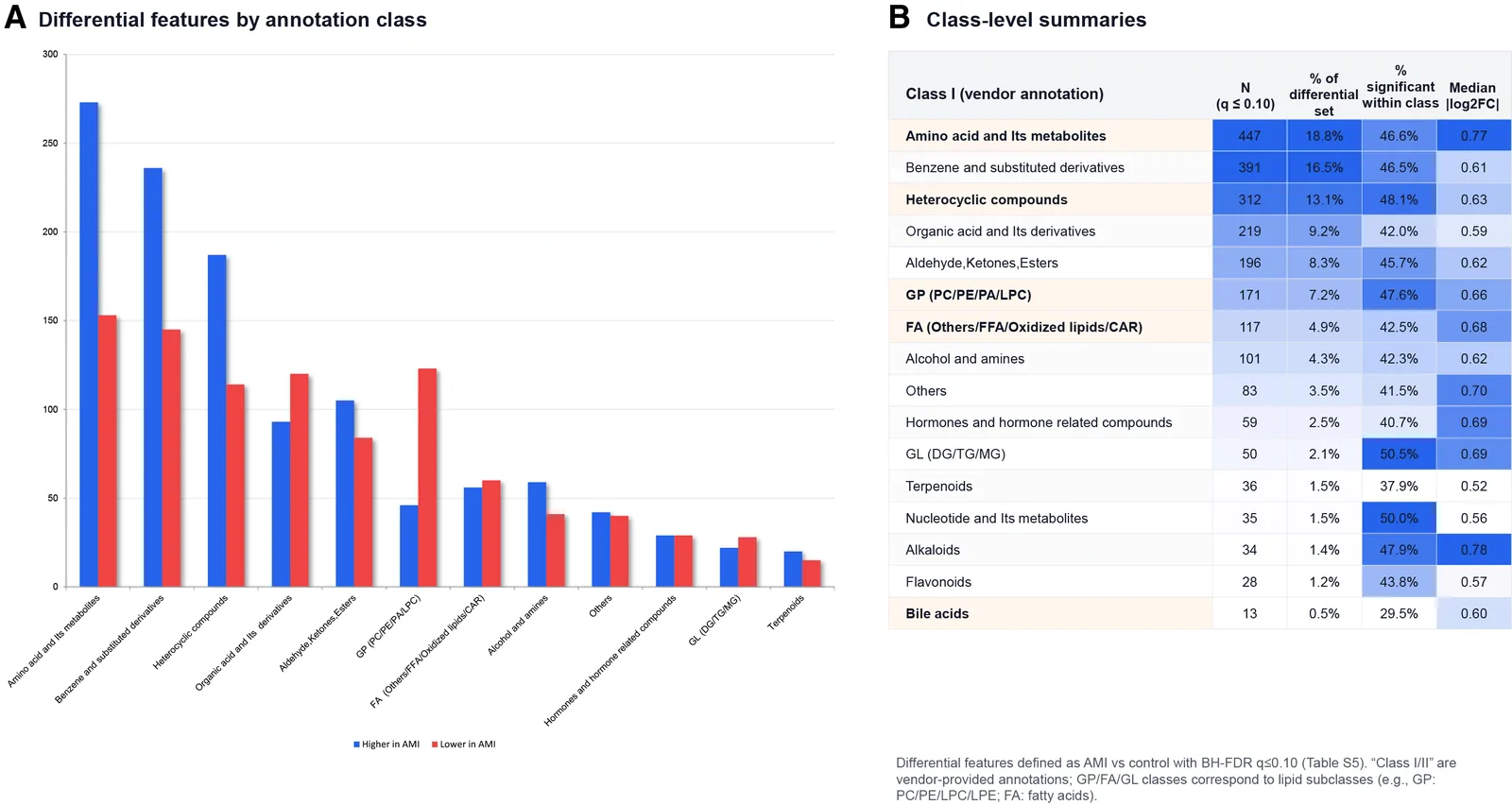
Descriptive class-level summary of AMI-healthy-control differential plasma metabolite features Numbers of significant plasma liquid chromatography-mass spectrometry (LC-MS) features are shown by Class I annotation and direction of change.

Untargeted plasma liquid chromatography-mass spectrometry (LC-MS) detected 5,271 features, of which 2,375 (45.1%) differed between AMI and healthy controls at BH-FDR q ≤ 0.10. Differential features were most frequent among amino acid and its metabolites, benzene and substituted derivatives, heterocyclic compounds, organic acid derivatives, glycerophospholipids (GP), and fatty acyls (FA), whereas bile acids were represented but numerically less common (Figure 7; Table S6).

We then tested whether these systemic shifts tracked archaeal-centered stool signals by correlating archaeal functional ROI scores with individual plasma LC-MS features and prespecified cardiometabolic metabolite families. No archaeal ROI-metabolite association survived BH-FDR q ≤ 0.10 in either group (Table S7). Thus, although plasma LC-MS captured a broad AMI-associated metabolome shift, the current paired stool-plasma dataset did not provide FDR-robust evidence linking archaeal stool ROI signals to circulating metabolite readouts.

### AMI severity associations

**Figure 8:**
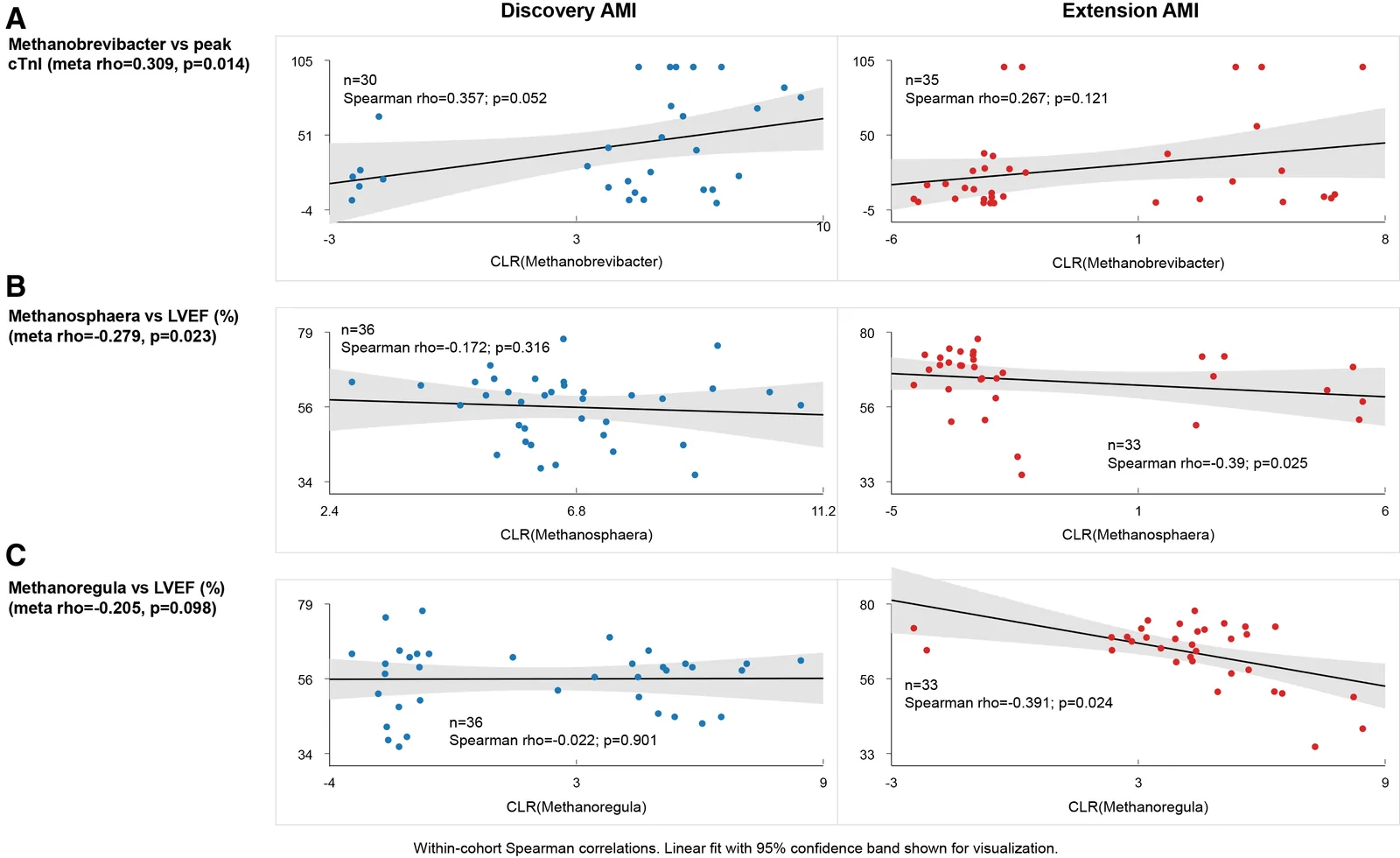
Exploratory post hoc archaeal taxa-AMI severity associations Candidate archaeal genera were related to AMI severity markers across two AMI sets. (A) *Methanobrevibacter* versus peak cardiac troponin I (cTnI). (B) *Methanosphaera* versus left ventricular ejection fraction (LVEF). (C) *Methanoregula* versus LVEF. Analyses were exploratory *post hoc* clinical anchoring analyses.

To clinically anchor archaeal signals highlighted by the discovery and extension analyses, we performed exploratory post hoc severity analyses of candidate archaeal genera against peak cTnI and left ventricular ejection fraction (LVEF), yielding six taxon-endpoint tests (Table S8). After BH-FDR correction across these six tests, *Methanobrevibacter* showed a positive association with peak cTnI (ρ_meta = 0.309, 95% CI, 0.065–0.518; *p* = 0.014; q = 0.069; I^2^ = 0.0%), whereas *Methanosphaera* showed an inverse association with LVEF (ρ_meta = −0.279, 95% CI, −0.488 to −0.040; *p* = 0.023; q = 0.069; I^2^ = 0.0%) (Figures 8A and 8B; Table S8).

By contrast, *Methanoregula* showed an inverse association with LVEF in the extension AMI set (ρ = −0.391; *p* = 0.024) but not in the discovery AMI set (ρ = −0.022; *p* = 0.901), and the meta-analysis did not reach FDR-level support (ρ_meta = −0.205, 95% CI, −0.426 to 0.039; *p* = 0.098; q = 0.196; I^2^ = 58.4%) (Figure 8C; Table S8). Age/sex/BMI-adjusted sensitivity analyses showed a similar direction for *Methanobrevibacter*-peak cTnI (adjusted ρ_meta = 0.291, 95% CI, 0.056 to 0.495; *p* = 0.016; q = 0.094) and *Methanosphaera*-LVEF (adjusted ρ_meta = −0.255, 95% CI, −0.468 to −0.014; *p* = 0.038; q = 0.115) (Table S8).

## Discussion

This archaeal-centered, four-domain metagenomic study shows that the gut archaeome in AMI is most informative as a state-dependent ecological signal, rather than as a set of strongly differential taxa. This point matters because cardiovascular microbiome research has largely been organized around bacteria-centric taxonomic shifts and metabolite-linked mechanisms, especially along the TMA/TMAO and PAGln axes.^1^^,^^2^^,^^3^^,^^4^^,^^5^^,^^6^ In contrast, our data indicate that archaeal signals in AMI are expressed primarily through cross-kingdom organization: the sign of A-B correlations, the domain in which rewiring concentrates, and the appearance of CAD→AMI transition edges. The study therefore reframes the archaeome not as a rare or weakly informative component of the gut microbiome but as a useful anchor for describing how multi-kingdom gut ecology changes across chronic coronary disease and acute infarction.

The first major finding is the directional shift in the AB layer. In the discovery cohort, significant AB correlations were predominantly positive in healthy controls but predominantly negative in AMI. This is biologically plausible in light of what is known about gut methanogens. Human-associated archaea, especially methanogenic lineages, consume H_2_/CO_2_ or methylated compounds and participate in interspecies hydrogen transfer, thereby influencing fermentation thermodynamics and redox balance within bacterial communities.^8^^,^^9^^,^^10^^,^^11^^,^^14^^,^^15^^,^^16^^,^^17^^,^^31^^,^^32^ Under relatively stable conditions, positive AB coupling is consistent with shared substrate ecology and syntrophic organization. A predominance of negative coupling in AMI instead suggests repartitioning of hydrogen-, acetate-, or methyl-compound niches during the acute coronary state. The taxon-level pattern supports that interpretation: AMI-specific negative edges involved archaeal lineages representing distinct methanogenic strategies, whereas the control state retained edges involving taxa adapted to acetate- or methyl-compound-linked niches. Taken together, these data argue that AMI perturbs how archaeal guilds are embedded within bacterial metabolism, rather than simply increasing or decreasing archaeal abundance.

A second important finding is that AMI-associated remodeling is not restricted to bacteria. In the binary AMI-healthy-control comparison, differential correlation was concentrated in the AF layer. This is notable because fungi are still often treated as background components in gut metagenomic studies, despite accumulating evidence that fungal communities can shape mucosal immunity and broader ecosystem behavior in inflammatory settings.^18^^,^^19^^,^^20^^,^^21^^,^^33^ Our findings extend that principle into cardiovascular disease: archaeal remodeling in AMI appears to occur within a genuinely multi-kingdom context, with fungal-associated rewiring representing the most extensive component of the discovery-cohort contrast. At the same time, the extension cohort retained few fungal genera under the prespecified thresholds, so direct three-state validation of this fungal-rich signal was underpowered. The most balanced interpretation, therefore, is that AF rewiring is a prominent discovery-cohort feature that now requires deeper mycobiome resolution or larger validation cohorts. More broadly, because the discovery and extension cohorts used different profiling workflows and retained different feature sets, cross-cohort convergence should be interpreted at the domain-layer level rather than as direct replication of individual taxa or edges. This distinction is summarized in Table 6.

The extension cohort adds a distinct and arguably more novel layer to the story. Severe CAD did not behave like a simple midpoint between angiographically normal controls and AMI. Instead, it represented a low-connectivity AB state, whereas AMI showed selective re-coupling and partner replacement. Some AB edges exhibited monotonic attenuation across CON→CAD→AMI, consistent with a chronic severity-linked weakening of baseline ecological coupling. But that was only part of the picture. Direct CAD–AMI testing identified a small set of sign-flip transition edges, including archaeal-virome co-variation edges. This distinction is conceptually important because it separates two processes that binary AMI-control studies usually conflate: a background of chronic ecological decoupling associated with severe atherosclerotic disease, and a more abrupt, event-linked reorganization that becomes visible only when severe CAD is explicitly included as an intermediate state.

The archaeal-virome co-variation layer showed disease-state enrichment across analyses. In the discovery cohort, archaeal-virome co-variation was more prominent in AMI than in healthy controls. In the extension cohort, three of the five CAD→AMI transition edges were A-V co-variation edges, all showing sign reversal between CAD and AMI. These findings should be interpreted as ecological archaeal-virome co-variation rather than direct AV interaction or A-V host linkage. Because formal viral host assignment was not performed, the observed A-V sign flips cannot distinguish between bacterial-phage–mediated community remodeling, archaeal virus-related biology, and shared host-state perturbation. Resolving these alternatives will require viral genome reconstruction, host-linking analyses, and perturbation models.^24^^,^^25^^,^^26^^,^^34^

The functional ROI analyses sharpen this ecological interpretation. Methanogenesis-linked scores showed little cross-domain connectivity, whereas the TMAO-linked electron-transfer score showed broader and more coherent positive coupling. This matters because the TMA/TMAO axis remains one of the clearest mechanistic links between the gut microbiome and cardiovascular disease.^1^^,^^2^^,^^3^^,^^6^^,^^35^ We do not interpret the stool TMAO_ET score as a direct measure of *in vivo* TMAO flux. Because discovery and extension scores were operationalized using MetaCyc pathways and a KO-based proxy, respectively, they were used for within-cohort ecological interpretation rather than direct quantitative cross-cohort comparison. Rather, the score appears to function as an annotation-based marker of a cardiometabolically relevant ecological niche, one that tracks broader multi-kingdom organization more clearly than generic methanogenesis readouts. That contrast is itself informative: archaeal functional signals are not interchangeable, and pathway-linked ecological summaries may be more revealing than broad methanogenesis proxies.

One apparently negative result—the disappearance of significant archaeal-module edges after conditional modeling—is also informative. In compositional, high-dimensional microbiome data, marginal correlations often capture broad ecosystem gradients and indirect structure, whereas stringent conditional inference removes much of that shared embedding.^27^^,^^28^^,^^29^^,^^30^^,^^36^^,^^37^ The fact that archaeal-module edges did not survive permutation control therefore suggests that the archaeal signal in AMI is largely community-embedded rather than densely pairwise. This does not weaken the central story; it refines it. The archaeome here appears less like an isolated driver and more like a sensitive participant in a larger ecological state.

The clinical anchoring analyses support that view. *Methanobrevibacter* showed a direction-consistent positive association with peak cTnI, and *Methanosphaera* a direction-consistent inverse association with LVEF, across two independently processed AMI sets. These findings do not establish causality, but they suggest that some archaeal-centered ecological states track host phenotypes linked to myocardial injury and systolic dysfunction. By contrast, the absence of FDR-robust archaeal ROI-metabolite associations in plasma indicates that archaeal-centered stool signals are more readily detected as local gut ecosystem states than as broad circulating correlates at the current sample size. That boundary condition is important: it suggests that archaeal-centered signatures may be more useful as markers of gut ecological organization than as direct plasma surrogates. Related cardiovascular metabolomics work has identified a putative oxylipin signature in patients with atrial fibrillation and coronary heart disease.^38^

These clinical anchoring analyses place the archaeal-centered network signal in a translational but appropriately bounded context. Rather than functioning as diagnostic substitutes, archaeal-centered ecological features address a different question: whether the acute coronary state is accompanied by a measurable host-microbiome configuration that may stratify microbial ecosystem context, recovery biology, or susceptibility to subsequent remodeling. Their associations with peak cTnI and LVEF provide an initial clinical bridge, but incremental prognostic value remains untested. Future cohorts with serial stool sampling and adjudicated outcomes should evaluate whether these features add discrimination, calibration, risk reclassification, or decision-curve benefit when integrated with conventional AMI models.

The same restraint applies mechanistically. Because AMI samples represented the first post-PCI bowel movement, the observed network patterns may reflect both AMI-associated biology and the accompanying early guideline-directed treatment and inpatient state. Acute inpatient illness can alter gastrointestinal motility, barrier function, and microbial ecology.^39^ Directional stability after covariate adjustment (Table 5) supports robustness to measured covariates but does not establish temporal ordering or causality. Because no post-discharge serial stool samples were collected, the present study cannot determine whether the observed remodeling is transient or persistent. Longitudinal sampling after discharge will be required to distinguish acute hospitalization-associated changes from sustained ecological alterations. Importantly, the virome-associated transition pattern also points to a forward-looking translational opportunity. Although the present data do not define viral hosts, viral functions, or causal phage-bacteria-archaea relationships, they suggest that the virome may represent an actionable ecological layer rather than a passive background signal. This concept is biologically plausible because gut phages can influence bacterial turnover, community stability, and metabolite output; experimental phage-perturbation studies have also shown that gut microbial communities and metabolite profiles can be reshaped *in vivo*.^24^^,^^25^^,^^26^^,^^34^^,^^40^^,^^41^ The A-V co-variation edges identified here should therefore be viewed as starting points for viral host-linking, functional annotation, and perturbation studies. Such studies will be needed to determine whether virome-associated remodeling reflects bacterial-phage–mediated community remodeling, archaeal virus-related biology, or broader host-state ecological coupling, and whether it is a reproducible marker—and eventually a modifiable component—of post-AMI gut ecosystem recovery.

An archaeal-centered, four-domain gut metagenomic framework indicates that AMI is characterized less by archaeal differential abundance than by cross-kingdom network remodeling. In particular, severe CAD appears to define a low-connectivity background state, whereas AMI is associated with an A-V co-variation pattern at the CAD-AMI transition. These findings extend bacteria-centric cardiovascular microbiome models and identify candidate archaeal taxa, functional signals, and transition edges for future validation. The prominent discovery AF rewiring signal warrants validation in larger cohorts with deeper mycobiome resolution.

### Limitations of the study

Several limitations should be acknowledged. The study is observational and cross-sectional. Because AMI samples represented the first post-PCI bowel movement, the observed network patterns may reflect both AMI-associated biology and the accompanying early guideline-directed treatment and inpatient state; these components cannot be separated in the present design. The discovery and extension cohorts used different metagenomic workflows and retained different feature sets, so cross-cohort comparison is limited to higher-level domain patterns rather than direct replication of individual taxa or edges. No post-discharge serial stool samples were available, precluding assessment of whether the observed remodeling is transient or persistent. Formal viral host assignment was not performed, and fungal resolution in the extension cohort was limited. Within these boundaries, the study identifies a coherent set of state-associated ecological patterns and candidate transition features for longitudinal and workflow-harmonized validation.

## Resource availability

### Lead contact

Requests for further information and resources should be directed to and will be fulfilled by the lead contact, Boqia Xie (dr.boqiaxie@mail.ccmu.edu.cn).

### Materials availability

This study did not generate new unique reagents.

### Data and code availability

De-identified stool metagenomic abundance data, derived network summaries, plasma LC-MS data, clinical covariate tables, functional ROI inputs, supplementary analysis tables, and analysis workflow scripts supporting the reported analyses are publicly available on Zenodo at https://doi.org/10.5281/zenodo.21633831. Any additional information required to reanalyze the data reported in this paper is available from the lead contact upon request.

## Acknowledgments

This work was supported by the 10.13039/501100001809National Natural Science Foundation of China (grant no. 6257070948) and the Shijingshan District Public Hospital Development Project (grant no. Shigongyifa 2025-1).

## Author contributions

Conceptualization, Y.Y., W.W., C.W., Y.Z., and B.X.; methodology, N.S., Z.Z., Y.Z., C.W., and B.X.; investigation, Y.Y., W.W., C.W., W.L., Q.S., and M.W.; writing – original draft, Y.Y., W.W., and C.W., writing – review and editing, W.L., Q.S., M.W., Y.Z., and B.X.; funding acquisition, Y.Z. and B.X.; resources, N.S. and Z.Z.; supervision, Y.Z. and B.X.

## Declaration of interests

The authors declare no competing interests.

## Declaration of generative AI and AI-assisted technologies in the writing process

During the preparation of this work, the authors used DeepSeek to improve the readability and English language of the manuscript. After using this tool, the authors reviewed and edited the content as needed and take full responsibility for the content of the published article.

## STAR★Methods

### Key resources table

**Table:** 

REAGENT or RESOURCE	SOURCE	IDENTIFIER
**Biological samples**		

Human stool samples	Beijing Chaoyang Hospital, Capital Medical University	NA
Human plasma samples	Beijing Chaoyang Hospital, Capital Medical University	NA

**Critical commercial assays**		

E.Z.N A.® Stool DNA Kit	Omega Bio-Tek	D4015-01
biowest Agarose	biowest	BY-R0100
Axygen® AxyPrep DNA Gel Extraction Kit	Axygen Biosciences	AP-GX-250
NEBNext Ultra II DNA Library Prep Kit	New England Biolabs	E7645L
DNBSEQ-T7RS High-throughput Sequencing Reagent Set	MGI	WLC26024
Qubit™ dsDNA HS Assay Kit	Thermo Fisher Scientific	Q32854
FS Pro DNA Lib PrepKit V2	ABclonal	RK20275
AFTMag Stool/Soil DNA Extraction Kit(Prepackaged)	ABclonal	RK30158
AFTMag NGS DNA Clean Beads	ABclonal	RK20257
Unique Dual index for Illumina Set A (48 indices)	ABclonal	RK21624
Unique Dual index for Illumina Set B (48 indices)	ABclonal	RK21625
QUBIT 1X dsDNA HS Assay Kit	ABclonal	RK30141

**Deposited data**		

De-identified data and analysis workflow scripts	This paper	Zenodo: https://doi.org/10.5281/zenodo.21633831

**Software and algorithms**		

R	R Foundation for Statistical Computing	https://www.r-project.org/

### Experimental model and study participant details

#### Human participants and study design

This multi-campus, hospital-based study was conducted at Beijing Chaoyang Hospital, Capital Medical University, across the Dongdaqiao, Changying, and Shijingshan campuses. We used a two-stage design comprising a discovery cohort and an independent prospective extension cohort. The discovery cohort included patients with AMI and healthy controls without known cardiovascular disease. For the present archaeal-centered analysis, the primary microbiome set was restricted to participants with complete stool shotgun metagenomic profiles across all four microbial domains—archaea, bacteria, fungi, and viruses—yielding 36 AMI patients and 46 healthy controls. A paired stool–plasma subset from the same discovery cohort (AMI *n* = 23; healthy controls *n* = 43) was analyzed separately for microbiome–metabolome coupling.

The extension cohort was prospectively enrolled and angiography-defined, comprising angiographically normal controls (CON, *n* = 22), patients with severe coronary artery disease without prior myocardial infarction (CAD, *n* = 29), and patients with AMI (*n* = 35). AMI participants in the extension cohort were selected from the available recruitment pool to improve balance in key clinical variables where feasible, including age, body mass index (BMI), diabetes status, and smoking. Baseline demographic and clinical characteristics of both cohorts are summarized in Table 1.

Across cohorts, exclusion criteria included inflammatory bowel disease, recent gastrointestinal surgery, active malignancy, chronic liver dysfunction, chronic inflammatory or autoimmune disease, and systemic antibiotic exposure within 2 months before sampling. The study was conducted in accordance with the Declaration of Helsinki and was approved by the institutional review board of Beijing Chaoyang Hospital (2025-ke-315). Written informed consent was obtained from all participants. The overall study design and analytic workflow are summarized in Figure 1.

#### Ethics approval and consent to participate

This study was conducted in accordance with the Declaration of Helsinki. The study protocol was reviewed and approved by the Ethics Committee/Institutional Review Board of Beijing Chaoyang Hospital, Capital Medical University (approval no. 2025-ke-315). All participants provided written informed consent prior to sample collection.

### Method details

#### Sample collection and processing

Fresh stool samples were collected in sterile tubes containing stabilizing buffer and stored at −80°C until metagenomic profiling. For AMI participants, stool samples were collected after percutaneous coronary intervention (PCI) and represented the first bowel movement after the procedure. Samples were collected within 72 h whenever feasible; this window accommodated delayed bowel movements in some patients related to strict bed rest after the acute event and intervention. Fasting or diet status at the time of stool passage was not restricted. AMI-related inpatient medications, including dual antiplatelet therapy and other clinically indicated therapies, were administered according to guidelines.^42^ In the extension cohort, stool samples from CAD and angiographically normal participants were collected within 24 h around coronary angiography.

For the paired stool–plasma subset, peripheral venous blood was collected on the same day as stool sampling. Plasma was separated by centrifugation and stored at −80°C until untargeted liquid chromatography–mass spectrometry (LC-MS) analysis.

#### Metagenomic profiling strategy and cross-cohort inference

To balance multi-kingdom breadth with domain-specific resolution, the discovery cohort was profiled using marker-based read profiling (MetaPhlAn4), whereas the prospective extension cohort used an assembly/contig-based profiling strategy to support genome-resolved characterization in the three-state design. Using distinct profiling strategies provided complementary workflow-level evidence for higher-level ecological patterns, but was not interpreted as feature-level replication.

Because profiling strategy and reference databases can yield different retained feature universes—and centered log-ratio (CLR) values are defined relative to the included feature set—we did not pool taxonomic CLR values across cohorts. All association and network analyses were performed within cohort, and cross-cohort synthesis relied on directionally concordant effects and, where applicable, fixed-effect meta-analysis of Fisher-z–transformed correlations rather than assuming absolute CLR comparability across pipelines.

#### Cross-domain preprocessing and feature screening

Before downstream association analyses, we applied prespecified, domain-agnostic curation to reduce obvious non-gut contamination and cross-domain name leakage. In the discovery cohort, we removed archaeal genera considered implausible gut residents or extreme-environment taxa (*Haloferax, Haloarcula, Halorubrum, Halobacter, Halogeometricum, Sulfolobus, Thermoproteus, Pyrococcus, Thermococcus, Nitrosopumilus*) and fungal genera unlikely to represent gut-resident signals (*Botrytis, Babesia*). In the extension cohort, we additionally removed archaeal genera flagged as likely non-gut/contaminant signals in this dataset (*Nitrososphaera, Methanocaldococcus, Haloplanus*). To prevent cross-domain leakage driven by identical genus labels, when the same genus name appeared in both the archaeal table and a non-archaeal table, we retained the archaeal entry and removed the duplicate from the non-archaeal table.

To respect domain-specific compositional constraints, all compositional preprocessing was performed within each domain separately. For each sample and each domain, genus profiles were total-sum scaled to obtain within-domain relative abundances, a pseudo-count was added (ε = 1 × 10^−6^), and CLR transformation was applied across genera within that domain:

$$\text{CL}R_{\text{ij}}=\log\left(R_{ij}+\varepsilon \right)-\frac{1}{p}\sum_{k=1}^{p}\log\left(R_{kj}+\varepsilon \right)$$

Where *R*_*ij*_ is the within-domain relative abundance of genus *i* in sample *j* and *p* is the number of retained genera in that domain. Domain-specific CLR matrices (archaea, bacteria, fungi, viruses) were then aligned by sample ID and used for all downstream within- and cross-domain analyses.

To reduce sparsity and stabilize inference, we applied prespecified abundance/prevalence screening within each cohort (pooled across clinical groups in that cohort). Genera were retained if relative abundance was ≥1 × 10^−4^ in ≥10% of samples. Prevalence thresholds were then applied using presence (relative abundance >0): archaeal genera were retained if present in ≥10% of samples in the pooled cohort dataset, whereas bacterial, fungal, and viral genera were required to be present in ≥20% of samples. Because bacterial genera substantially outnumbered other domains, bacterial genera were ranked by variance of pooled CLR values and the top 300 were retained for cross-domain screening; all fungal and viral genera passing the above filters were retained.

Viral taxonomic profiles were parsed at the genus level from cohort-specific taxonomic tables. Viral genera passing the prespecified partner-domain filters (relative abundance ≥1 × 10^−4^ in ≥10% of samples and prevalence ≥20%) were retained for virus-specific CLR transformation. Formal viral host assignment was not performed; A–V edges are reported as archaeal–virome co-variation.

#### Archaeal functional ROIs

We prespecified three annotation-derived archaeal functional regions of interest (ROIs) capturing (i) hydrogenotrophic methanogenesis, (ii) acetoclastic methanogenesis, and (iii) trimethylamine N-oxide (TMAO)-linked electron-transfer annotations. ROI scores were computed per sample from stool functional abundance profiles and log1p-transformed to stabilize scale. Specifically, in the discovery cohort, ROI scores were derived from MetaCyc pathway abundance profiles as.

(i) methanogenesis_H2_score = log (1 + Abundance [METHANOGENESIS-PWY]);
(ii) methanogenesis_acetate_score = log (1 + Abundance [METH-ACETATE-PWY]);
(iii) TMAO_ET_score = log (1 + Abundance [PWY0-1355] + Abundance [PWY0-1578]).

PWY0-1355 and PWY0-1578 correspond to formate-to-trimethylamine N-oxide electron transfer and hydrogen-to-trimethylamine N-oxide electron transfer, respectively.

In the extension cohort, methanogenesis_H2_score and methanogenesis_acetate_score were computed from the corresponding MetaCyc pathways using the same log1p definitions. Because pathway-level MetaCyc outputs for TMAO electron transfer were unavailable in the extension cohort, TMAO_ET_score was derived from a prespecified KEGG Orthology (KO) -based proxy comprising TMAO reductase/electron-transfer annotations in the KO output:

$$\text{TMAO}\_\text{ET}\_\text{score}=\log\left(1+\sum_{KO\in S_{\text{TMAO}}}Abundance\left[KO\right]\right)$$

Where *S*_TMAO_ = {*K*03532, *K*07811, *K*07812, *K*07821}. TMAO_ET results were interpreted within cohort-specific annotation frameworks.

#### Genus-level archaeal-centered cross-domain screening

Within each cohort, archaeal-centered cross-domain associations were screened separately within each clinical group using two-sided Spearman correlations computed on CLR-transformed abundances. For each group, we correlated each archaeal genus (and each archaeal functional ROI score) with each partner-domain genus in bacteria, fungi, and viruses using the prespecified candidate pools. Multiple testing was controlled using Benjamini–Hochberg FDR (BH-FDR) within each group × domain-pair family: archaeal–bacterial (A–B), archaeal–fungal (A–F), and archaeal–virome (A–V), and edges were considered significant at *q* ≤ 0.10.

Differential-correlation analysis in the discovery cohort and the extension-cohort within-state, CAD–AMI transition, and three-state trajectory analyses are described below.

#### Module-level nodes and archaeal-anchored conditional networks

To evaluate whether archaeal cross-domain signals persisted after accounting for broader within-domain community structure, we reduced each partner domain (bacteria, fungi, viruses) to within-domain co-abundance modules and tested archaeal-module associations under a conditional (near-partial) framework. Module construction was performed on pooled CLR profiles from the discovery-cohort stool-only four-domain intersection (AMI + healthy controls) to obtain fixed module definitions independent of case/control labels.

Within each partner domain, we computed within-domain Pearson correlations between genera on CLR-transformed values, converted correlations to a dissimilarity matrix (1−r), and applied hierarchical clustering. The number of modules was selected using a gap-statistic criterion subject to a minimum module size of 10 genera. Each module was summarized by its module eigengene (the first principal component across member genera). Exact clustering parameters, final module counts and sizes, eigengene variance explained, bootstrap stability metrics, and module memberships are provided in Table S1.

To quantify robustness, we performed bootstrap resampling of individuals (200 resamples), reconstructed modules under the same procedure, and evaluated Jaccard similarity-based stability for each original module. Stability metrics were used descriptively to annotate module robustness and were not imposed as hard inclusion/exclusion criteria.

Archaeal genera were retained as individual nodes (i.e., not clustered) to preserve archaeal resolution. For each group (AMI and healthy controls) and each bipartite relationship (A–B, A–F, A–V), the node set comprised all screened archaeal genera and the corresponding partner-domain module eigengenes. To approximate conditional (near-partial) cross-domain associations under collinearity and high dimensionality (*p*≫*n*), we estimated ridge-residual near-partial correlations on CLR-transformed data. For each node *X*_*j*_, we fitted a ridge regression (*α* = 1.0; intercept included; predictors z-standardized) of *X*_*j*_on all other nodes in the same relationship-specific node set and retained the residual vector ${\hat{\varepsilon }}_{j}$. The near-partial association between nodes *j* and *k* was defined as the Spearman correlation between residuals,

$$\rho jk=\text{Spearman}\left({\hat{\varepsilon }}_{j},{\hat{\varepsilon }}_{j}\right)$$

Edge-level inference used a two-sided Freedman–Lane permutation procedure (*B* = 120 permutations) applied to the residual-based association, with Benjamini–Hochberg FDR control at *q* ≤ 0.10within each prespecified group × relationship family.

#### Differential correlation (“rewiring”) analysis in the discovery cohort (AMI vs. healthy controls)

To quantify AMI–healthy-control differences in archaeal-anchored cross-domain correlation structure on a common node set, we performed edge-wise differential correlation testing using group-specific Spearman correlations on CLR-transformed abundances. For each domain pair (A–B, A–F, A–V), tested edges were defined on the fixed node set consisting of screened archaeal genera and the corresponding partner-domain module eigengenes defined under “Module-level nodes and archaeal-anchored conditional networks”. Correlations were estimated within AMI and within healthy controls using pairwise complete observations (the subscript “control” denotes healthy controls in the discovery cohort), for each edge, the effective sample sizes were denoted *n*AMI and *n*control.

For each edge, Spearman correlations *ρ*AMI and *ρ*control were Fisher *r*-to -*z* transformed as $z\left(\rho \right)=\frac{1}{2}\ln\left(\frac{1+\rho }{1-\rho }\right)$, yielding *z*_AMI_ = *z*(*ρ*_AMI_) and *z*_control_ = *z*(*ρ*_control_). Group differences were evaluated by

$$z_{\Delta }=\frac{z_{\text{AMI}}-z_{\text{control}}}{\sqrt{\frac{1}{n_{\text{AMI}}-3}+\frac{1}{n_{\text{control}}-3}}}$$

Two-sided *p*Δ values were obtained from the standard normal distribution and Benjamini–Hochberg FDR was controlled at *q*Δ ≤ 0.10 within each domain-pair family. Edges were highlighted as differentially correlated (“rewired”) only when they met both the prespecified statistical and effect-size criteria: *q*Δ ≤ 0.10 and ∣Δ*ρ*∣ ≥ 0.30, where Δ*ρ* = *ρ*_AMI_−*ρ*_control_ (Spearman *ρ* on CLR values).

Extension cohort: within-state screening, CAD–AMI transition testing, and three-state trajectory summaries (CON–CAD–AMI).

In the extension cohort (CON, severe CAD, AMI), archaeal-centered cross-domain patterns were evaluated in three complementary analyses designed to separate severity-linked attenuation from state-dependent CAD–AMI transitions.

#### Within-state archaeal-centered screening

Within each clinical state separately, we computed two-sided Spearman correlations on CLR-transformed abundances between each screened archaeal genus and each partner-domain genus for A–B, A–F, and A–V. Multiple testing was controlled using Benjamini–Hochberg FDR at *q* ≤ 0.10 within each state × domain-pair family, yielding state-specific sets of significant archaeal-anchored edges.

#### CAD–AMI transition edges

To identify state-dependent rewiring at the acute transition, we directly contrasted CAD versus AMI correlations on the shared screened feature set for each domain pair. For each archaeal-partner pair, CAD and AMI correlations were compared using Fisher’s r-to-z framework (as above), with two-sided *p* values adjusted by Benjamini–Hochberg FDR at *q*diff ≤ 0.10 within each domain-pair family. Transition edges were defined as those meeting *q*diff ≤ 0.10; sign reversals (opposite-signed *ρ* in CAD vs. AMI) were additionally summarized as an interpretable subset of transition behavior.

#### CON–CAD–AMI trajectory and network-state summaries

To characterize severity-like patterns beyond pairwise testing, we summarized edge trajectories across ordered clinical states (CON–CAD–AMI) for edges that were significant in at least one state (at *q* ≤ 0.10). “Severity-like” trajectories were defined as monotonic changes in correlation strength ∣*ρ*∣ across the three states without sign reversal. In parallel, we reported state-level network summaries including (a) significant edge counts by domain pair and sign, and (b) archaeal degree distributions (number of significant edges incident to each archaeal genus) to capture hub structure and hub switching across states.

#### Sensitivity analyses

To evaluate sensitivity to measured clinical imbalance, genus-level CLR profiles were rank-residualized against age, sex, BMI, smoking, drinking, diabetes, hyperlipidemia, and hypertension, followed by recomputation of archaeal-centered correlations and transition-Δ directions on residualized profiles both in the discovery cohort and in the extension cohort.

For headline edge-level findings, bootstrap and permutation stability analyses were performed. Participants were resampled within disease group 2,000 times to estimate Δρ direction stability and percentile 95% confidence intervals (CI). Disease labels were also permuted 2,000 times to estimate empirical significance for Δρ. These analyses were applied to discovery AMI–healthy-control rewired edges and extension CAD–AMI transition edges.

#### Exploratory AMI severity associations

Exploratory AMI severity analyses were post hoc and candidate-driven. Candidate archaeal genera were selected from the archaeal-centered network analyses, and clinical endpoints were restricted to peak cardiac troponin I (cTnI) and left ventricular ejection fraction (LVEF) as markers of myocardial injury and systolic function. This yielded six candidate taxon–endpoint tests. Within each AMI set, Spearman correlations were calculated and synthesized by fixed-effect meta-analysis of Fisher-z–transformed correlations. BH-FDR correction was applied across the six meta-analysis *p* values. For covariate-adjusted sensitivity analyses, variables were rank-residualized against age, sex, and BMI before correlation analysis, followed by the same fixed-effect meta-analysis framework.

#### Untargeted plasma LC-MS metabolomics

Untargeted plasma metabolomics was performed using LC-MS/MS in both positive and negative ion modes. Plasma samples were thawed on ice, vortexed for 10 s, and 50 μL of each sample was transferred into a labeled centrifuge tube. Samples were extracted with 300 μL of 20% acetonitrile/methanol extraction solution containing internal standards, vortexed for 3 min, and centrifuged at 12,000 rpm for 10 min at 4°C. The supernatant was transferred to a new tube, incubated at −20°C for 30 min, centrifuged again at 12,000 rpm for 3 min at 4°C, and the final supernatant was transferred for LC-MS analysis.

Chromatographic separation was performed on a Waters ACQUITY Premier HSS T3 column (1.8 μm, 2.1 × 100 mm). Mobile phase A was 0.1% formic acid in water, and mobile phase B was 0.1% formic acid in acetonitrile. The column temperature was 40°C, the flow rate was 0.4 mL/min, and the injection volume was 4 μL. Mass spectrometry was performed on an AB TripleTOF 6600 platform in ESI+ and ESI− modes.

Pooled QC samples were prepared by mixing study-sample extracts and inserted throughout the analytical run, approximately once every 10 study samples. QC assessment included total ion chromatogram overlap, blank-sample assessment, QC-sample correlation, internal-standard stability, CV distribution, and PCA-based process monitoring.

Raw MS data were converted to mzXML format using ProteoWizard and processed using XCMS for peak extraction, peak alignment, and retention-time correction. Peak areas were corrected using an SVR-based signal-correction procedure.

Metabolite features were annotated using experimental spectral databases, integrated public databases, predicted spectral libraries, and metDNA-based annotation. Features were retained after annotation and QC filtering using a comprehensive annotation score >0.5 and QC CV < 0.30. Positive- and negative-ion-mode features were merged by retaining the feature with the higher annotation level and lower QC CV where applicable.

Feature-level analyses were performed on individual LC-MS features. Metabolite-family summaries were based on Class I annotations and were used for descriptive class-level interpretation.

#### Microbiome–metabolome coupling

In the paired stool–plasma subset, untargeted plasma LC-MS generated semi-quantitative feature intensities. Analyses were performed within AMI and within healthy controls separately. Within each group, metabolite features were retained if they had ≥70% non-missing values; remaining missing values were imputed as one-half of the minimum positive value for that feature within the corresponding group. Intensities were then log_10_-transformed, and features with zero variance after preprocessing were removed.

Microbiome–metabolome coupling was tested by Spearman correlations between microbiome exposures (archaeal taxa on CLR scale, archaeal functional ROI scores, and archaeal-anchored module-level nodes) and plasma metabolite readouts, including both individual metabolite features and metabolite family scores. Metabolite families were defined using vendor-provided Class I annotations, and family scores were computed per sample as the mean log_10_ intensity across metabolites in the family. Multiple testing was controlled using Benjamini–Hochberg FDR at *q* ≤ 0.10, applied within each metabolite family and within prespecified cardiometabolic panels (including TMAO-related metabolites, phenylacetylglutamine (PAGln), bile acids and related lipids).

### Quantification and statistical analysis

All statistical tests were two-sided, with BH-FDR controlled within prespecified comparison families at q ≤ 0.10 unless stated otherwise. The q ≤ 0.10 threshold was used as a prespecified exploratory FDR threshold for high-dimensional archaeal-centered network screening. Sensitivity inspection using q ≤ 0.05 yielded sparser edge sets but did not alter the main domain-layer interpretation; q ≤ 0.10 findings were therefore interpreted as exploratory ecological signals rather than confirmatory edge-level discoveries. Analyses were performed in R; key packages included WGCNA/dynamicTreeCut (module construction), ppcor (partial correlation utilities), and tidyverse/ggplot2 (data handling and visualization).

## Supplemental information

Table S1. Module construction, eigengene properties, stability metrics, and genus membership

Table S2. Extension-cohort within-state archaeal-centered cross-domain correlations

Table S3. Archaeal-bacterial correlation trajectories across angiographically normal controls, severe CAD, and AMI

Table S4. Extension-cohort archaeal functional region of interest analyses (A) ROI score differences across CON-CAD-AMI. (B) Within-state ROI-genus association summary (BH-FDR q ≤ 0.10).

Table S5. Bootstrap and permutation stability analyses for discovery-cohort rewiring and CAD-acute myocardial infarction transition edges

Table S6. Differential plasma LC-MS features between AMI and healthy controls

Table S7. Archaeal functional region of interest and plasma metabolite associations in the paired stool-plasma subset (A) Prespecified cardiometabolic metabolite-family scores (B) Top nominal feature-level associations within the predefined metabolite panel

Table S8. Exploratory associations of candidate archaeal genera with peak cardiac troponin I and left ventricular ejection fraction across AMI sets (A) Primary fixed-effect meta-analysis (B) Age/sex/BMI-adjusted sensitivity analysis
